# Match-Related Statistics Differentiating Winning and Losing Teams at the 2019 Africa Cup of Nations Soccer Championship

**DOI:** 10.3389/fspor.2022.807198

**Published:** 2022-02-25

**Authors:** Alliance Kubayi, Paul Larkin

**Affiliations:** ^1^Department of Sport, Rehabilitation and Dental Sciences, Faculty of Science, Tshwane University of Technology, Pretoria, South Africa; ^2^Institute for Health and Sport, Victoria University, Melbourne, VIC, Australia

**Keywords:** team performance, tactics, statistics, counter-attacks, shots

## Abstract

This study investigated game-related statistics differentiating the winning and losings teams of matches during the 2019 African Cup of Nations (AFCON) soccer tournament. The sample consisted of 38 games, with the data obtained from the InStat Scout platform. Data were analyzed using mean (M), SD, effect size (ES), structure coefficients (SCs), and the Wilcoxon signed-rank test. The results showed that the winning teams performed significantly better than the losing teams in terms of shots (M = 12.13, SD = 4.67, Z = −2.26, ES = 0.62), shots on target (M = 5.05, SD = 2.54, Z = −4.22, ES = 1.13), and shots from counter-attacks (M = 2.24, SD = 1.42, Z = −2.48, ES = 0.57). Shots on target (SC = 1.22), shots (SC = −0.73), fouls (SC = 0.60), total passes (SC = 0.44), and yellow cards (SC = −0.32) presented the highest discriminatory power. These findings highlight the key match performance variables which influence the game results and may assist coaches in developing and implementing team strategies to improve the likelihood of winning the AFCON championship.

## Introduction

The Africa Cup of Nations (AFCON) is the largest continental soccer championship organized under the auspices of the Confederation of African Football (CAF). The first competition was held in 1957, with the following four countries competing: Egypt, Sudan, Ethiopia, and South Africa. Since 1968, the tournament has been held every 2 years, with the number of teams increasing to eight in 1968 and 12 in 1992. With the return of South Africa to the African soccer competitions in 1996, the tournament expanded to 16 teams; however, Nigeria withdrew from the competition due to political issues, thereby reducing the number of teams to 15 (Confederation of African Football, 2014; Kubayi and Toriola, 2020a). In 2019, the number of teams expanded to 24 in order to give more countries an opportunity to participate in the championship. The AFCON is rated as the most popular tournament on the continent, watched by millions of people in every African country (Njororai, 2019).

Despite the popularity of soccer in Africa (Kubayi et al., 2015), there is limited information on the match performance of national teams in the African competitions (Njororai, 2019; Kubayi and Toriola, 2020b). Currently, match analysis research in soccer is focused mainly on European competitions (Mao et al., 2016; Zhou et al., 2018). For instance, previous studies have reported that the winning teams had higher averages than the losing teams in terms of performance variables such as shots, shots on target, shots from open play, shots from counter-attacks, total passes, accurate passes, crosses, through balls, corners, dribbles, and ball possession (Lago-Peñas and Lago-Ballesteros, 2011; Lago-Peñas et al., 2011; Liu et al., 2015). In addition, the losing teams committed more fouls and received more yellow cards than their winning counterparts in Europe (e.g., Spanish La Liga, UEFA Champions League). This shows that the winning teams are primarily effective in variables related to goal scoring and retaining ball possession, which is a style of play associated with success in both the European and the international competitions (Collet, 2013; Kubayi and Toriola, 2020b).

To date, the available published data on key performance indicators in African soccer has focused on ball possession in domestic competitions (Kubayi and Toriola, 2019). For example, Kubayi and Toriola (2019) reported that the losing teams had higher ball possession than the winning teams in the South African Premier Soccer League. However, a limitation of the findings was the lack of analysis related to the potential impact of match performance indicators that can lead to team success. Due to the evolving nature of soccer, as well as the need for analysts and coaches to be informed of the current in-game performance trends, it is essential to reassess not only the possession of the ball during a match but other possible game-related statistics that may contribute to effective performance (Oberstone, 2009; Araya and Larkin, 2013).

From a continental perspective, there is little knowledge of the main performance indicators that may influence the African match results. Therefore, understanding the key match statistics is crucial, as the continent's football structure is relatively less developed and needs more scientific information (Zhou et al., 2018) to potentially inform future tactical decisions and coaching processes (Kubayi and Larkin, 2019). By understanding the current trends in team performance, coaches may be able to devise team tactics to maximize the chances of winning the competitions. Therefore, this study aimed to investigate game-related statistics that differentiated the winning and losing teams during the 2019 AFCON soccer tournament.

## Methods

### Sample, Data Source, and Variables

Data of all 52 matches played during the 2019 AFCON soccer championship were obtained from the InStat Scout platform and subsequently analyzed. The competition consists of 24 teams divided into six groups of four teams. Each team plays with the other three in their group once, with three points earned for a win, one point for a draw, and zero for a loss. In every group, the winners and runners-up, as well as the four best third-placed teams, qualify for the knock-out stage of the tournament (i.e., 16 teams). The knock-out stage starts with the round of 16, followed by quarter-finals, semi-finals, third-place playoff, and the final. For the purpose of this study, the 14 matches which ended in draws were excluded from the analyses. Therefore, the final sample consisted of 38 games played during the competition. Ethical clearance was granted by the institution of the lead author's ethics committee.

Match statistics consisted of variables related to goal scoring (i.e., shots, shots on target, and shots from counter-attacks), passing and organizing (i.e., passes, percentage of accurate passes, percentage of ball possession, dribbles, and percentage of successful dribbles), and defending (i.e., tackles, percentage of tackles won, fouls, and yellow cards). The operational definitions of these variables are provided in a previous study (Lago-Peñas et al., 2011; Liu et al., 2015; Mao et al., 2016).

### Reliability Testing

The inter-observer test was used to assess the reliability of the data. Two independent soccer analysts, who were not part of the research team, coded each of the four randomly selected matches (i.e., 10.5% of the sample). Thereafter, the two data sets were computed to assess the level of agreement. Cronbach's α coefficient was used to assess the reliability of the match statistics. The alpha values ranged from 0.71 to 1.00. A value above 0.70 is considered to be within the acceptable limits and reliable (Taber, 2018).

### Statistical Analysis

Data were reported as means (Ms) and SDs. A Wilcoxon signed-rank test was used to compare the match statistics between the teams that won and lost. A significance level was set at *p* ≤ 0.05, and the effect size (ES) was used to assess the magnitude of the differences in the mean scores of the studied variables. The ES values were categorized as follows: trivial (<0.20), small (0.20–0.59), moderate (0.60–1.19), large (1.20–2.00), and very large (>2.00) (Batterham and Hopkins, 2006). Finally, a discriminant function analysis was carried out to identify the game-related statistics that differentiated winners and losers. The structural coefficient (SC) was used to identify the performance variables that best contributed to the discrimination between winners and losers. The SC value ≥ |0.30| was considered to have a significant contribution between the groups (Tabachnick and Fidell, 2001). Statistical analyses were conducted using IBM SPSS Version 26.

## Results

Table 1 shows differences in the game-related statistics between the winning and losing teams during the 2019 AFCON soccer tournament. The findings highlighted that the winners performed significantly better than the losers for the following variables: shots (M = 12.13, SD = 4.67, Z = −2.26, *p* = 0.02, ES = 0.62), shots on target (M = 5.05, SD = 2.54, Z = −4.22, *p* = 0.001, ES = 1.13), and shots from counter-attacks (M = 2.24, SD = 1.42, Z = −2.48, *p* = 0.01, ES = 0.57). The winning teams had higher averages, although not significant, than the losing teams for defensive-related variables such as ball recovery and the tackles won.

**Table 1:** Match statistics between the winning and losing teams in the 2019 African Cup of Nations (AFCON) soccer tournament.

	**Win**	**Lose**	***Z***	**Sig**.	**ES**
**Goal scoring**					
Shots	12.13 ± 4.67	9.47 ± 3.93	−2.26	0.02*	0.62
Shots on target	5.05 ± 2.54	2.63 ± 1.66	−4.22	0.00*	1.13
Shots from counter attacks	2.24 ± 1.42	1.42 ± 1.48	−2.48	0.01*	0.57
**Passing and organizing**					
Passes	421.39 ± 96.74	393.58 ± 77.51	−0.97	0.33	0.31
Accurate passes (%)	80.97 ± 4.25	79.84 ± 3.15	−1.21	0.23	0.30
Ball possession (%)	51.32 ± 8.13	48.71 ± 8.12	−0.98	0.33	0.32
Dribbles	27.08 ± 7.31	25.63 ± 6.42	−0.92	0.36	0.21
Successful dribbles (%)	14.92 ± 5.44	14.24 ± 4.34	−0.84	0.40	0.14
**Defending**					
Ball recoveries	50.58 ± 7.68	48.34 ± 7.67	−1.76	0.09	0.29
Tackles	32.82 ± 6.94	32.42 ± 8.55	−0.04	0.97	0.05
Tackles won (%)	56.89 ± 8.39	54.32 ± 10.69	−1.19	0.24	0.27
Fouls	19.16 ± 5.68	17.79 ± 4.59	−1.05	0.29	0.26
Yellow cards	1.37 ± 1.05	1.79 ± 0.88	−1.64	0.10	0.43

* *Significant at p < 0.05*.

Table 2 presents the discriminant function structure coefficients of the winning and losing teams. The findings showed that the discriminant function was statistically significant (*p* < 0.05) and correctly classified 78.9% of the cases. Match statistics variables, such as shots on target (SC = 1.22), shots (SC = −0.73), fouls (SC = 0.60), total passes (SC = 0.44), and yellow cards (SC = −0.32) had the highest discriminatory power.

**Table 2:** Discriminant function, SC, between the winning and losing teams in the 2019 AFCON soccer tournament.

**Variables**	**SC**
Shots on target	1.22*
Shots	−0.73*
Fouls	0.60*
Total passes	0.44*
Yellow cards	−0.32*
Shots from counter attacks	0.25
Ball possession (%)	−0.25
Successful dribbles (%)	0.20
Tackles won (%)	0.17
Tackles	0.13
Accurate passes (%)	0.09
Ball recoveries	−0.06
Dribbles	0.04
Eigenvalue	0.60
Wilks' lambda	0.62
Canonical correlation	0.61
Chi-squared	31.71
Significance	0.00
Reclassification (%)	78.9

* *SC discriminant value > |0.30|*.

## Discussion

The main purpose of this study was to investigate the game-related statistics differentiating the winning and losing teams during the 2019 AFCON soccer tournament. The findings highlighted that all goal-scoring variables significantly discriminated between the teams that won and lost. The current study substantiates the notion that the winning teams perform better in relation to the goal-scoring performance variables compared to the losing teams (Lago-Peñas et al., 2011). Specifically, the findings showed that the winning teams had significantly greater total shots and shots on target than their counterparts. However, the magnitude of the difference was larger on the shots on target than on the total shots. Further, shots on the target had a higher discriminatory power (SC = 1.22) than the total shots (SC = −0.73). These findings are consistent with those of previous studies, which confirmed the shooting quality (i.e., accuracy) rather than the quantity that determines the match outcome in soccer (Yue et al., 2014; Liu et al., 2015; Varley et al., 2017; Kubayi and Larkin, 2020). Considering the high importance of shots on the target, coaches should consider developing training activities with a focus on shooting accuracy (Zhou et al., 2018).

In addition, it was found that the winning teams had significantly more shots from counter-attacks than the losing teams. Previous research has shown that the teams using the counter-attacking style of play tend to create more goal-scoring opportunities (Tenga et al., 2010a). This is attributed to the fact that a counter-attack quickly moves the ball into the opponent's final third of the field, which forces the defending team to immediately reorganize from an unorganized defensive structure (Kim et al., 2019). Therefore, such a style of play appears to generate a degree of imbalance in the opposition defense, which can result in goals being scored (Tenga et al., 2010b). Therefore, it is suggested that coaches should design the training programmes that encourage counter-attack play to gain a potential advantage over the opponents.

The findings from the current study also indicated that the losing teams received more yellow cards than those who won. The discriminatory power of yellow cards was SC = −0.32, indicating a negative impact on the team performance. This result corroborates with previous studies demonstrating that the number of yellow cards in the UEFA Champions League significantly discriminated between the winners and the losers (Lago-Peñas et al., 2011). Kubayi and Toriola (2020b) also confirmed that in the 2018 FIFA World Cup, the African teams were issued more yellow cards than their European counterparts, which compromised their performance. Further, the current study found that the winning teams had higher averages, albeit being nonsignificant than the losing teams for the defensive-related variables, such as ball recovery and the tackles won. These results are consistent with the previous studies, which found that successful tackles and prompt recovery of ball possession are related to team success (Almeida et al., 2014; Vogelbein et al., 2014; Liu et al., 2015). The present observations indicate that successful teams not only need to be effective in attacking but also must defend well in order to increase their chances of winning games.

The discriminant function analysis showed that the total passes had high discriminatory power. The descriptive analysis also indicated that the winning teams had a higher number of passes than those which lost. This is a key finding, as a greater number of passes may be an effective approach in building attacking a play and creating more opportunities to shoot at goal (Jones et al., 2004; Araya and Larkin, 2013). However, no significant differences were found between the winners and losers in terms of the accuracy of passes and ball possession. While a previous study has found that for the European teams, passing accuracy is strongly linked with holding on to the ball (Collet, 2013), for African matches, the ball possession does not define the successful team performance (Kubayi and Toriola, 2019). Bradley et al. (2014) also found that dominant teams in the European competitions have adopted a possession style of play, suggesting that they prefer to “control” the game by dictating the play, but if a team is unable to retain the ball possession, a “direct” style of play might be a more appropriate game tactic. This direct style quickly takes the ball into shooting positions, thereby possibly creating more goal-scoring opportunities (Kite and Nevill, 2017).

## Conclusion

The results of the current study highlighted the game-related statistics that influenced match results during the 2019 AFCON soccer competition. The findings showed that the winning teams had a greater number of total shots, shots on target, and shots from counter-attacks compared to the losing teams. Further, shots on target, shots, fouls, total passes, yellow cards, and shots from counter-attacks had the highest discriminatory power. These results may assist the African soccer coaches in tailoring match tactics and implementing training activities to increase the chances of winning matches during the AFCON championships.

## Data Availability Statement

The raw data supporting the conclusions of this article will be made available by the authors, without undue reservation.

## Author Contributions

Both authors listed have made a substantial, direct, and intellectual contribution to the work and approved it for publication.

## Conflict of Interest

The authors declare that the research was conducted in the absence of any commercial or financial relationships that could be construed as a potential conflict of interest.

## Publisher's Note

All claims expressed in this article are solely those of the authors and do not necessarily represent those of their affiliated organizations, or those of the publisher, the editors and the reviewers. Any product that may be evaluated in this article, or claim that may be made by its manufacturer, is not guaranteed or endorsed by the publisher.

## References

[B1] Almeida C. H. Ferreira A. P. Volossovitch A. (2014). Effects of match location, match status and quality of opposition on regaining possession in UEFA Champions League. J. Hum. Kinet. 41, 203–214. 10.2478/hukin-2014-0048 25114747 PMC4120454

[B2] Araya J. A. Larkin P. (2013). Key performance variables between the top 10 and bottom 10 teams in the English Premier League 2012/13 season. Hum. Mov. Health Coach Educ. 2, 17–29.

[B3] Batterham A. M. Hopkins W. G. (2006). Making meaningful inferences about magnitudes. Int. J. Sports Physiol. Perform. 1, 50–57. 10.1123/ijspp.1.1.50 19114737

[B4] Bradley P. S. Lago-Peñas C. Rey E. Sampaio J. (2014). The influence of situational variables on ball possession in the English premier league. J. Sports Sci. 32, 1867–1873. 10.1080/02640414.2014.887850 24786661

[B5] Collet C. (2013). The possession game? A comparative analysis of ball retention and team success in European and international football, 2007–2010. J. Sports Sci. 31, 123–136. 10.1080/02640414.2012.727455 23067001

[B6] Confederation of African Football (2014). The History of AFCON. Available online at: http://www.caf.com (accessed April 12, 2020).

[B7] Jones P. James N. Mellalieu S. (2004). Possession as a performance indicator in soccer. Int. J. Perform. Anal. Sport 4, 98–102. 10.1080/24748668.2004.11868295

[B8] Kim J. James N. Parmar N. Ali B. Vučković G. (. (2019). Determining unstable game states to aid the identification of perturbations in football. Int. J. Perform. Anal. Sport 19, 302–312. 10.1080/24748668.2019.1602439

[B9] Kite C. S. Nevill A. (2017). The predictors and determinants of inter-seasonal success in a professional soccer team. J. Hum. Kinet. 58, 157–167. 10.1515/hukin-2017-0084 28828086 PMC5548163

[B10] Kubayi A. Larkin A. (2020). Technical performance of soccer teams according to match outcome at the 2019 FIFA Women's World Cup. Int. J. Perform. Anal. Sport 20, 908–916. 10.1080/24748668.2020.1809320

[B11] Kubayi A. Larkin P. (2019). Analysis of teams' corner kicks defensive strategies at the FIFA World Cup 2018. Int. J. Perform. Anal. Sport 19, 809–819. 10.1080/24748668.2019.1660547

[B12] Kubayi A. Toriola A. (2019). The influence of situational variables on ball possession in the South African Premier Soccer League. J. Hum. Kinet. 66, 175–181. 10.2478/hukin-2018-0056 30988851 PMC6458569

[B13] Kubayi A. Toriola A. (2020a). Match performance indicators that discriminated between winning, drawing and losing teams in the 2017 AFCON soccer championship. J. Hum. Kinet. 72, 215–221. 10.2478/hukin-2019-0108 32269662 PMC7126255

[B14] Kubayi A. Toriola A. (2020b). Differentiating African teams from European teams: Identifying the key performance indicators in the FIFA World Cup 2018. J. Hum. Kinet. 2019:144. 10.2478/hukin-2019-0144 32774551 PMC7386154

[B15] Kubayi N. A. Coopoo Y. Morris-Eyton H. F. (2015). Job-related barriers encountered by football coaches in Gauteng Province of South Africa. Afri. J. Phys. Health Educ. Recreat. Dance 21, (Suppl.1), 160–166.

[B16] Lago-Peñas C. Lago-Ballesteros J. (2011). Game location and team quality effects on performance profiles in professional soccer. J. Sports Sci. Med. 10, 465–471. 24150619 PMC3737821

[B17] Lago-Peñas C. Lago-Ballesteros J. Rey E. (2011). Differences in performance indicators between winning and losing teams in the UEFA Champions League. J. Hum. Kinet. 27, 135–146. 10.2478/v10078-011-0011-3

[B18] Liu H. Gómez M. A. Lago-Peñas C. Sampaio J. (2015). Match statistics related to winning in the group stage of 2014 Brazil FIFA World Cup. J. Sports Sci. 33, 1205–1213. 10.1080/02640414.2015.1022578 25793661

[B19] Mao L. Peng Z. Liu H. Gómez M. A. (2016). Identifying keys to win in the Chinese professional soccer league. Int. J. Perform. Anal. Sport 16, 935–947. 10.1080/24748668.2016.11868940

[B20] Njororai W. W. S. (2019). Organizational factors influencing football development in East African countries. Soccer Soc. 20, 168–188. 10.1080/14660970.2017.1302931

[B21] Oberstone J. (2009). Differentiating the top English Premier League Football Clubs from the rest of the pack: identifying the keys to success. J. Quantitat. Anal. Sports 5, 1–27. 10.2202/1559-0410.1183

[B22] Tabachnick B. G. Fidell L. S. (2001). Using Multivariate Statistics, 3rd Edn. New York, NY: Harper Collins.

[B23] Taber K. S. (2018). The use of Cronbach's alpha when developing and reporting research instruments in science education. Res. Sci. Educ. 48, 1273–1296. 10.1007/s11165-016-9602-2

[B24] Tenga A. Holme I. Ronglan L. Bahr R. (2010a). Effect of playing tactics on goal scoring in Norwegian professional soccer. J. Sports Sci. 28, 237–244. 10.1080/02640410903502774 20391095

[B25] Tenga A. Holme I. Ronglan L. T. Bahr R. (2010b). Effect of playing tactics on achieving score-box possessions in a random series of team possessions from Norwegian professional soccer matches. J. Sports Sci. 28, 245–255. 10.1080/02640410903502766 20391096

[B26] Varley M. C. Gregson W. McMillan K. Bonanno D. Stafford K. Modonutti M.. (2017). Physical and technical performance of elite youth soccer players during international tournaments: influence of playing position and team success and opponent quality. Sci. Med. Football 1, 18–29. 10.1080/02640414.2016.1230676

[B27] Vogelbein M. Nopp S. Hokelmann A. (2014). Defensive transition in soccer – are prompt possession regains a measure of success? A quantitative analysis of German Fußball-Bundesliga 2010/2011. J. Sports Sci. 32, 1076–1083. 10.1080/02640414.2013.879671 24506111

[B28] Yue Z. Broich H. Mester J. (2014). Statistical analysis for the soccer matches of the first Bundesliga. Int. J. Sports Sci. Coach. 9, 553–560. 10.1260/1747-9541.9.3.553

[B29] Zhou C. Zhang S. Lorenzo Calvo A. Cui Y. (2018). Chinese soccer association super league, 2012–2017: key performance indicators in balance games. Int. J. Perform. Anal. Sport 18, 645–656 10.1080/24748668.2018.1509254

